# Response to Lewis et al. (2026): Clarifying the comparative characterization of EMPOWER in the ILIA study protocol

**DOI:** 10.1007/s00406-026-02331-w

**Published:** 2026-08-07

**Authors:** Stefan Leucht

**Affiliations:** Technical University of Munich, School of Medicine and Health, Department of Psychiatry and Psychotherapy, TUM University Hospital, Munich, Germany

Dear Editor,

We read the letter by Lewis et al. [1] with some surprise. It was never our intention to offend the EMPOWER research group, whose contributions to the field of digital relapse prevention in schizophrenia spectrum disorders are widely recognized and explicitly acknowledged in our protocol [2]. The comparative discussion in the ILIA protocol [2], compromising only twelve lines within a ten-page article, was written not as a critique of EMPOWER but as a means of delineating design differences between two related yet methodologically distinct interventions addressing a shared clinical problem. We consider scientific exchange and critique essential to the advancement of the field. We welcome this opportunity for clarification and are glad to address points where our original formulations were imprecise. For several other points, however, we would like to provide the reasoning that informed our original statements.

## "No validated questionnaire was used"

We acknowledge the publication by Palmier-Claus et al. [3] and accept that our original formulation may have been imprecise in not referencing this work. However, a careful reading of that paper reveals that it was explicitly framed as a proof-of-concept study, with the stated aim of assessing the feasibility and convergent validity of ambulatory smartphone self-report against PANSS and Calgary Depression Scale scores obtained before and after the one-week. The paper [3] reports on the psychometric properties of a symptom self-report battery administered via the ClinTouch platform, on which the EMPOWER publication was technically built.

A comparison of the item sets, as documented in the respective supplementary materials of both studies [3, 5], indicated limited recognizable overlap in item wording or number of core items between the two questionnaires. On this basis, a shared psychometric foundation was not apparent to us. We therefore could only identify a limited shared psychometric foundation between the questionnaire examined by Palmier-Claus et al. [3] and the questionnaire used in EMPOWER. Accordingly, our point was not that no prior work on smartphone-based symptom self-report existed. Rather, our statement referred specifically to the questionnaire used in the EMPOWER study. Based on the available documentation, we remain of the view that this specific questionnaire had not been fully validated as a psychometric instrument prior to its use.

## "The intervention did not provide a digital interface and feedback loop to the treating clinician and no joint action between the patient and the treating clinician was mandatory"

We recognized that the EMPOWER feasibility cRCT builds upon the technological infrastructure developed in Lewis et al. [4]. However, the EMPOWER publications [5–7] state explicitly:

*“****Changes in EWS were observable by the researchers***, *and responses were manual rather than automated. [.] Any detected increase meant that the* ***study research assistant advised the clinical team*** *of any significant change to the participant’s mental health.”* [6].

**Fig. 1:**
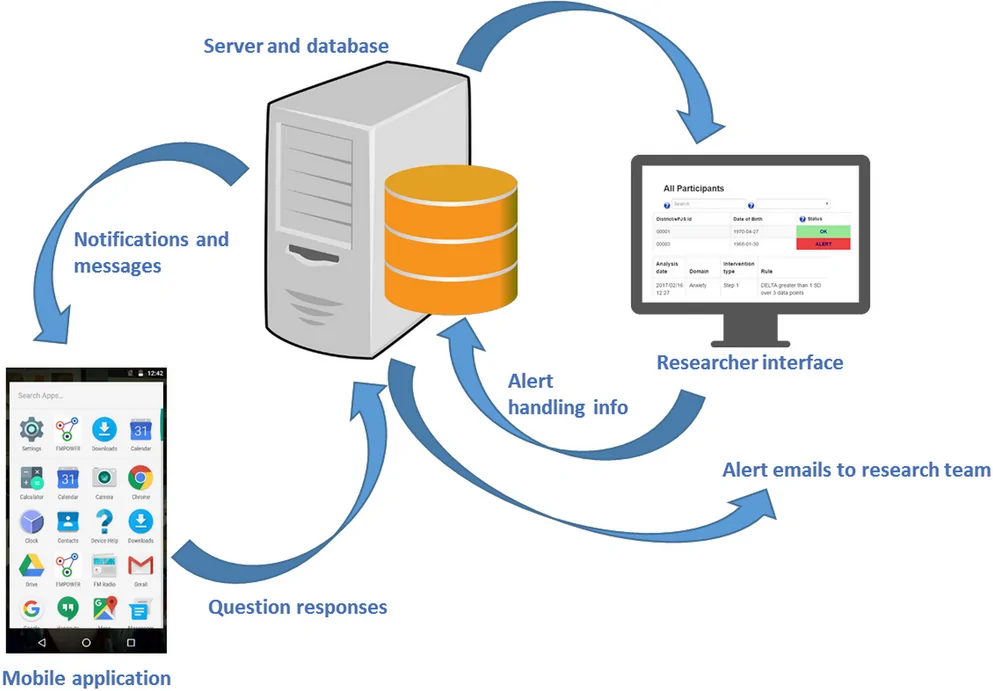
Visualization of the EMPOWER system as presented in [6], including the presentation of the “researcher interface”

*“The consequences of the ChIP [alert] are that the* ***research team***, *which includes a registered mental health nurse (in the United Kingdom)*,* clinical psychologists (United Kingdom and Australia)*,* and general psychologist (Australia only)*, ***is emailed*** *about the participant and that the participant’s status is set to alert and is highly* ***visible on a secure Web-based researcher interface***. *[…] In* ***response to ChIPs***,*** a member of the research team checks*** *in with the participant and based on the outcome of this triage assessment*,* they* ***can share an update with the participants’ care coordinator*** *who can*,* if indicated*,* escalate increased support to the participant from their local CMHS to reduce risk of relapse. These actions are agreed on a case-by-case basis*,* and* ***we did not constrain the service user’s treatment provider to respond*** *within a specific timeframe or indeed in a specific way.”* [7]

Notably, the authors themselves label this component a “*researcher interface*” [5–7], as depicted in Fig. 1. In the EMPOWER feasibility cRCT, the clinical team, as part of the research team, was the downstream endpoint of a researcher-triaged escalation chain, reached only after the research team had screened and filtered the incoming data.

Our protocol observation was therefore not incorrect, as we did not claim that EMPOWER lacked a digital interface per se, but that – in contrast to our ILIA study - it did not provide a ***direct feedback loop to the treating clinician***,*** as local treating clinicians*** did not have ***direct access*** to the patient-reported data and alerts (i.e., ChIPs). We acknowledge that in the study by Lewis et al. [4], the ClinTouch app was integrated into NHS Trust information and technology platforms, ***forwarding summary information to clinicians via the electronic health records*** (EHRs). Whether information was also directed into the respective EHRs in the EMPOWER study was not apparent to us from reading the EMPOWER publications [5–7].

In ILIA, an automated alert is sent simultaneously and directly to the patient’s personal ***local treating clinician*** and to the patient upon threshold exceedance on the EWSQ-10P. Documented clinician response within our web-based portal is a mandatory, protocol-specified step [2]. In the EMPOWER trial, information reached the clinical team only after screening and escalation by the research staff. This remains an important procedural distinction from the ILIA trial, which we sought to outline in the protocol’s discussion not as a criticism, but as a clarification of differences in study design and clinical implementation. Based on this reasoning, we do not agree with Lewis’ interpretation that our statement is inaccurate. Rather, we maintain that the distinction drawn in the protocol is scientifically justified and accurately reflects the different pathways through which clinically relevant information was transferred to the treatment team in the two studies.

## "The number of assessments during the 12-month intervention period appears to be quite burdensome"

Our formulation used deliberately hedged language (“*appears to be quite burdensome*”) [2] and was not an empirical claim about dropout causation. The observation concerned the volume of the daily monitoring schedule (up to 56 items per daily assessment in EMPOWER) as a potential challenge for routine implementation. We wish to emphasize once more that this observation was not intended as a criticism of the EMPOWER monitoring design per se. A daily, multi-item assessment schedule is a legitimate and potentially highly sensitive approach to symptom tracking, and we made no claim to the contrary. We, however, intended to contextualize our own design decision to implement weekly monitoring using ten early warning sign items [2]. This choice was made deliberately, with the aim of reducing participant burden in a long-term multicenter trial embedded in German routine clinical care. A retention rate of 94% is a meaningful indicator of participant engagement and speaks to the quality of the EMPOWER implementation. At the same time, retention under these closely supported feasibility conditions may not straightforwardly generalize to routine implementation or larger-scale trials, and the authors’ own call for a main trial with *n* = 500 participants [6] suggests that feasibility-phase findings warrant further validation before broader conclusions can be drawn.

## "The study did not focus on the assessment of hospitalization measures (e.g. days spent in hospital, time to hospitalization) as main indicators of effectiveness on relapse"

Lewis et al. [1] present hospitalization data as a rebuttal to our statement that EMPOWER did not focus on hospitalization measures “*as* ***main indicators of effectiveness*** *on relapse*” [2]. However, this does not address the point we made. We did not state that the EMPOWER trial failed to collect hospitalization data. Rather, we highlighted that hospitalization was not a primary endpoint of the trial.

EMPOWER was explicitly designed as a feasibility trial, with feasibility of the trial and feasibility, acceptability and usability of the intervention as its main outcomes. The publications describe relapse and fear of relapse as “candidate co-primary outcomes” [6, 7], indicating their role as potential outcomes for a future definitive effectiveness trial rather than endpoints for which the feasibility trial was powered. Accordingly, EMPOWER was not powered to detect differences in relapse or hospitalization outcomes:

*“Our main outcomes were establishing feasibility*,* acceptability*,* usability and safety*,* which were assessed in the intention-to-treat population which included all participants from each randomised center. We also included several exploratory clinical outcomes to aid in designing and planning a future definite clinical trial”* [5].

Our observation was only motivated by a design difference relevant to ILIA, in which hospitalization is a pre-specified primary outcome for which the trial is statistically powered [2].

## Criticisms on the ILIA design and a note on precise reading

The ILIA protocol operates under ethics approval from the TUM Ethics Committee (2024-242-S-KK) including clearly defined adverse event procedures [2]. Clinical safety is therefore addressed. The absence of EHR integration and formal carer involvement were deliberate design choices rather than design deficiencies. ILIA was primarily conceived as an initial evaluation of the clinical effectiveness of our digital early warning signs monitoring in German routine outpatient care. More extensive implementation features, including EHR integration and broader stakeholder involvement, are important considerations for future large-scale effectiveness and implementation trials. Design features shared with EMPOWER (e.g. 12 months follow-up period, relapse within the last 2 years) reflect well-established methodological standards in relapse prevention research, additionally acknowledged with explicit citation in our protocol [2].

A further concern raised in the letter relates to the impression that we had adopted scales from the EMPOWER trial without appropriate engagement. We would like to clarify that this does not reflect the actual course of events. Members of our group have been in direct contact with researchers involved in the EMPOWER trial, including Professor Andrew Gumley. As part of this exchange, we discussed the possibility of collaboratively validating the relevant FORSE scale in Germany. Importantly, our group offered co-authorship and a shared publication pathway for this work. This illustrates that our intention was not to copy or independently appropriate existing material, but rather to establish a transparent collaborative process and to benefit from mutual scientific exchange.

Against this background, we were surprised by the way this concern was raised in the letter. We would have welcomed direct communication at an earlier stage, as this could likely have clarified the issue and allowed for a more constructive mutual agreement. We believe that such dialogue is central to scientific debate, particularly in a field in which different groups ultimately share the same goal to develop and evaluate meaningful interventions for vulnerable clinical populations. Accordingly, we would like to emphasize that our group has in fact pursued the opposite of what was implied in the critique. We actively engaged with colleagues from the EMPOWER trial, sought collaboration, and aimed to contribute to a transparent validation process rather than to duplicate or appropriate existing work.

Finally, Lewis et al. [1] suggested that we have not read the EMPOWER publications carefully. This is an allegation we take seriously and have addressed throughout this response with detailed textual evidence. We invite the same standard of precision in return. Lewis et al. [1] state that the ILIA trial “borrowed” design features from the EMPOWER trial, including cluster randomization. This is incorrect as the ILIA trial uses individual 1:1 randomization, stratified by study center [2]. Contrary to EMPOWER, we have no cluster randomized controlled trial.

We thank Lewis et al. [1] for the opportunity to clarify these points and look forward to what both trials will contribute to the evidence base for digital relapse prevention in schizophrenia spectrum disorders.

## Data Availability

No datasets were generated or analysed during the current study.

## References

[1] Lewis S, on behalf of the EMPOWER trial group (2026) Letter to the Editor, Eur Arch Psychiatry Clin Neurosci. 10.1007/s00406-026-02291-1 42287360

[2] Hiller S, Emde L, Jais D et al (2025) The ILIA study: protocol for a randomized-controlled multicenter clinical trial on smartphone- and web-based relapse monitoring for patients with schizophrenia or schizoaffective disorder. Eur Arch Psychiatry Clin Neurosci 276:637–650. 10.1007/s00406-025-02089-7 40828423 PMC12953440

[3] Palmier-Claus JE, Ainsworth J, Machin M et al (2012) The feasibility and validity of ambulatory self-report of psychotic symptoms using a smartphone software application. BMC Psychiatry 12:172. 10.1186/1471-244X-12-172 23075387 PMC3502449

[4] Lewis S, Ainsworth J, Sanders C et al (2020) Smartphone-enhanced symptom management in psychosis: open, randomized controlled trial. J Med Internet Res 22:e17019. 10.2196/17019 32788150 PMC7453320

[5] Gumley AI, Bradstreet S, Ainsworth J et al (2022) The EMPOWER blended digital intervention for relapse prevention in schizophrenia: a feasibility cluster randomised controlled trial in Scotland and Australia. Lancet Psychiatry 9:477–486. 10.1016/S2215-0366(22)00103-1 35569503

[6] Gumley AI, Bradstreet S, Ainsworth J et al (2022) Digital smartphone intervention to recognise and manage early warning signs in schizophrenia to prevent relapse: the EMPOWER feasibility cluster RCT. Health Technol Assess 26(27). 10.3310/hlze047 35639493 PMC9376801

[7] Gumley A, Bradstreet S, Ainsworth J et al (2020) Early Signs Monitoring to Prevent Relapse in Psychosis and Promote Well-Being, Engagement, and Recovery: Protocol for a Feasibility Cluster Randomized Controlled Trial Harnessing Mobile Phone Technology Blended With Peer Support. JMIR Res Protoc 9(1):e15058. 10.2196/15058 31917372 PMC6996736

